# Round the Hospitals

**Published:** 1918-08-03

**Authors:** 


### ROUND THE HOSPITALS.

The first-fruits of the efforts now in progress for the adequate endowment of the College of Nursing are to be observed in the announcement, which will be found in our advertisement columns, of three studentships of 100 guineas each offered by the College to trained nurses for a year's course of instruction at the Household and Social Service Department of King's College for Women, University of London. This valuable course has been planned with wisdom and practical insight. It gives exact training in the various branches of knowledge requisite for ensuring the well-being of households, small and large. But it does far more than this. By full and varied courses of lectures on social science it puts the students into close relation with the forces now stirring for the regeneration of the people, and can hardly fail to induce in them that larger outlook hitherto only to be found here and there in British training-schools for nurses.

Under the direction of the able tutors at Campden Hill the course can be specially modified or enlarged to meet the requirements of the student's future career. The immediate object of the year's course at King's College for Women is to fit nurses for holding the post of sister-tutor or instructress in a nurse-training school. There is a greater demand for such tutors than can at present be filled, and the work of the schools is hampered by the fact that the ordinary training course does not and cannot give the specialised training requisite for the nursing tutor. We look forward to the time when to hold the certificate of this year's training will be valued among the matron's list of qualifications for office.

Candidates for the studentships offered by the College of Nursing ought not to be discouraged by the prospect of the examination. The conditions may be obtained from the Secretary of the College of Nursing, 6 Vere Street, W. 1, on or before August 17, 1918. These candidates for the first studentships of the kind offered in the history of British nursing will not be subjected to competitive examination, nor even, we may surmise, be compelled to obtain a given number of marks. The object of the examination is to reveal ability, and for this purpose the general paper with short essay on a professional or other topic is well chosen. The general paper casts its net far and wide, not to expose ignorance, but to discover knowledge and the ability to express it. The essay gives opportunity for looking into the student's mind and gauging some of the tendencies it displays. What the examiners look for is promise rather than fulfilment.

An excellent article on "The Future of Nursing" in the *Daily Telegraph* of July 26 acquaints its readers with the forward movement in the nursing world associated with the College of Nursing and the Nation's Tribute Fund. The *Daily Telegraph* is always remarkably well informed on nursing subjects, and it is a great advantage that one of the leading daily papers should study to put before the public in true perspective the matters which concern the nursing profession, its difficulties, its aims, and its triumphs. There is undoubtedly no profession which has such unique claims on the public sympathy, and even its peculiarly technical struggles after a standardised training should command the sympathy of all who will benefit by improvement in nurse-training—that is to say, all the classes and all the masses.

The fifth year of war opens at the very core of the conflict for a free world. Any doubt which may have hung over the root causes of the war and the aims of the principal combatant countries has been dissipated during the past few months. It is true that the Kaiser and his press industriously repeat, parrot-like, the call of the Allies to battle for the peace of the world. But it is only too evident that by a Kaiser peace is meant a prelude to the reign of force, crushing the weaker nationalities into submission—the German way of rule by fear and hatred. Gradually the issue has been narrowed. One by one among the Allies national causes for mutual suspicion and jealousy have faded away, and to-day, at the outset of the fifth year, the countries which have taken liberty and justice for their watchword grip more closely together in the will to win an enduring peace. It has taken a long time to make the nation and its nurses realise that peace is only to be won by conflict. The tendency to catch at every straw which might restrain the downward, trampling rush of the warriors to the field of Armageddon is still strong in our midst, but hope that a way may be found short of the surrender of all that is noblest among us to sheer combat slowly fades.

When the nation kneels on the appointed Day of Intercession to pray "Thy will be done," the prayer must be no formal act of submission to untold horrors dealt out by a mysterious fate, but an act of self-dedication by each and all to carry into effect that will for peace on earth proclaimed by angels to be the end and aim of Christ's kingdom on earth. There are few besides the nurses among women who have been admitted to the inmost secrets of what war is costing humanity. There are none who are spending themselves more entirely than nurses in the desperate task of alleviating its woes. There are none who have followed more closely in the steps of the Master through the past four years. On this fatal anniversary the watchword of the whole Nursing Service rings out clear and strong, "Thy will be *done*—Thy will to heal the wounded be carried into effect, Thy will to compensate the bruised in mind and the maimed and sorrowful be fulfilled through our hands. Thy will for peace on earth be achieved through the medium of constant faith and effort."

Among recent instances of heroism in the nursing world which we are proud to collect is that

**ROUND THE HOSPITALS—(*continued*).**

of Miss Fraser, a British nurse who is also a motor ambulance driver, and is attached to the 4th Army. While transporting the wounded she was herself twice wounded seriously by an aerial torpedo which wrecked her car. She made her way on foot to the nearest hospital and reported to the medical officer that there were wounded in her car, refusing succour for her own injuries until they had been brought in. Miss Fraser has been awarded the Legion of Honour and the Croix de Guerre.

St. Bartholomew's Hospital has taken a wise and timely step in the direction of satisfying the legitimate aspirations of the V.A.D. The governors have decided to admit to training a limited number of nursing members of Voluntary Aid Detachments, and special military probationers who have satisfactorily completed not less than two consecutive years' work in a military or an auxiliary hospital. The hospital certificate of training will be granted after the passing of the final examination on the completion of three years' training, the fourth year of the usual course being excused. Regular probationers are now received at St. Bartholomew's Hospital at the age of twenty-one. The concession made by St. Bartholomew's Hospital and by the Nightingale Home at St. Thomas's in reducing the training of what may be called war probationers to three years opens the way for admission to the nursing profession, under very easy conditions, of women likely to prove very valuable additions to it. Already they have given evidence of a devotion to their country and to the nursing art which deserves recognition in the training-school. Their after-career will be watched with much interest.

The Royal Free Hospital has become possessed of a welcome extension to its midwifery department by taking over from the Duchess of Marlborough her Maternity Home in Endsleigh Street, Tavistock Square. The financial pressure on the Home has of late become acute, and it could not have met a better fate than to be absorbed into the sphere of the Royal Free Hospital, where extended opportunities of midwifery training are urgently needed. A considerable extension of the Home will take place under the new management as soon as circumstances will admit.

The decoration of the Royal Red Cross, First Class, was conferred by His Majesty on July 25 on Sister Margaret Percival, Q.A.I.M.N.S., and on Assistant-Matron Mary Chapman and Sister Annie MacLeod of the Reserve. The following were made Associates of the Order: Sister Jane Galloway, Sister Frederica Roche, Matron Margaret Mullally, Sister Aggie Durward, Sister Elizabeth Weller, Matron Louisa Denton, Matron Jessie Elms, Matron Elsie Gale, Matron Lilian Gibbon, Assistant-Matron Edith Draper, Sister Frances Eager, Sister Phœbe Ellwood, Sister Clara Evans, Sister Anne Farmer, Sister Jane Gordon, Sister Ida Gould, Sister Lavinia Green, Staff-Nurse Norah Fitzgerald, Miss Esther Edwards, Miss Beatrice Evans; also Matron Ethel Graham and Sister Ruby Cockburn, of the British Red Cross Society.

With further reference to the School of Massage in Cape Town which we foreshadowed in a recent issue of The Hospital, we have ascertained that the Cape Hospitals Board has agreed to establish a training-school, and the Medical Council at the Cape is making arrangements to issue a certificate after examination. The Hospitals Board lays it down that the teacher of massage at any such school must have the teaching certificate of the Incorporated Society of Trained Masseuses of London. The standard of the certificate must be up to the requirements of the Incorporated Society and the examiner appointed by the Medical Council should be approved by this Society also. The object aimed at is that the holders of these certificates, when they are recognised and approved, will be qualified to practise in any part of the British Empire. The salary of the teacher of massage will not be less than £20 a month, with board and lodging, and she will preferably hold a certificate of general training as well as that of the Incorporated Society of Trained Masseuses.

The Hampshire County Nursing Association has taken the sensible course of appealing to women through the correspondence columns of the local press to offer themselves for training as village nurse-midwives. The scarcity of probationers for this branch of work is due, we are convinced, to the ineptitude of the county organisation in failing to make known their needs among the women likely to respond and prove good midwives. Every village midwife ought to be a recruiting sergeant for her Association. If supplied with short readable papers setting forth the conditions of service she could assist better than anyone else to draw in new workers, for she could allay the timidity and want of self-confidence which afflicts dwellers in the country and holds them back from the career of nursing.

Ulster nurses have been making their mark in France, first as a separate unit under control of the Service de Santé, and then with an American unit, and have won golden opinions. They have lately been working near the Western Front, and have shared in the disagreeable experience, common to many nursing sisters recently, of assisting in the evacuation of their station under shell-fire. The Germans fired on them while removing the stretcher cases. They are appealing for more Ulster nurses to join them, and for money to enable them to carry on as a separate unit under the Service de Santé. Applicants must apply to the matron, Samaritan Hospital, Belfast, and fluent French is one of the necessary qualifications.

The work of the Scottish Women's Hospital in Corsica, to which island they transferred their activities after the removal of the hospital from Salonika, is very little known in this country, and Sister Mary Walker had a deeply interested audience at Keighley on July 23, when she gave particulars

**ROUND THE HOSPITALS—(*continued*).**

of it. There are 10,000 Serbian refugees in Corsica under the care of the Scottish Women's Hospitals, and already 100 babies have been born, of which three died. The opportunity of instilling sound notions on the rearing of infants is not being wasted, and much practical training in sanitation and personal hygiene is given among the camps, which may bear good fruit later on when these pioneers in the paths of cleanliness are restored to their homes. Already the Serbian nation is planning to found sanitary systems on the model of France and England, and the Maternity Centre on approved modern lines is an indispensable adjunct to a higher civilisation.

---

THE sinking on July 15 of H.M. Transport *Barunga*, formerly the German *Sumatra*, conveying invalided Australian troops back to their own country, gave occasion for the display of remarkable coolness on the part of both soldiers and nursing sisters. Largely owing to the sang-froid of all concerned, there were no casualties. Had everything been minutely rehearsed beforehand the discipline could not have been better. Within the short half-hour after the torpedo struck the vessel until it sank everyone on board behaved as though it were in the ordinary routine of their duties to be cast into the ocean. The helpless cripples, of whom there were about a dozen, awaited rescue with well-justified confidence. There were four nursing sisters on board, including Matron Blundell, who, though severely shaken by the explosion, "set a splendid example." All, in fact, were "just lovely," in the words of an eyewitness. They were thrown to the floor in the hospital cabins, but speedily resumed labours among the sick and assisted in their rescue. They have added fresh lustre to the Australian Military Nursing Service.

---

THE rapid rise in cost of nursing uniforms has received attention from the Willesden Urban District Committee, which has been allowing its nursing staff £8 per annum for uniform. The Health Committee having reported that this sum is now insufficient to cover the cost, and that in some cases uniform is not worn by the staff, has instructed the medical officer to purchase uniforms at a cost not exceeding £12, the uniform to be worn by all. We confess to some doubt as to whether the medical officer be the person best fitted to deal with this matter. The chief nurse would probably lay out the money to better advantage. There is no doubt that the present high cost of materials is a very serious matter for institutions which provide uniform, and the Willesden estimate of new requirements at half as much again is by no means excessive.

---

AT the Quarterly Executive Committee of the County Councils Association, held on July 24 at the Middlesex Guildhall, Westminster, the report of the Nursing Sub-Committee dealing with the co-operation of nursing associations with the County Councils in maternity and child-welfare schemes was read and discussed. It was finally resolved that "This committee, while agreeing that County Nursing Associations through their affiliated associations may render valuable service to County Councils in connection with health visiting and allied work (including school nursing), are of opinion that such assistance should be rendered by them as agents under the supervision of the County Councils." This is a good deal better than the wording suggested by the sub-committee, which ruled that "County Nursing Associations, as such, should take no part in the administrative work or in supervising the efficiency of the work done." Such a display of jealousy on the part of the County Councils Association would have been deplorable under existing circumstances. The important thing, after all, is to get to work and save the babies.

---

IN the London County Council schools dental inspection is proceeding on admirable lines. We note in the recently issued list of inspections during the Michelmas term the names of several women dentists. Short addresses on the care of the teeth are to be given by the inspecting dentist at each visit to the mothers of the children, specially invited for the occasion. In this way the inspection becomes a real educative force in each district, and the immeasurable loss and discomfort which attend neglected teeth will be checked in the surest way by demonstration.

---

WE have been asked to extend the time for entering for the nursing leaflets competition outlined in our last issue in order to give an opportunity to some who are out of England to send in their names. The conditions are as follows: 1. The leaflets must be short, from 1,000 to 2,000 words. 2. Leaflets should each deal with one branch, and one only of nursing work, as, for instance, private nursing, fever nursing, village nursing, midwifery, and so on. Competitors may send in one or more leaflets. 3. Leaflets should be written for outsiders, and explain in easy language the conditions of training and future prospects of women who qualify for the branch of nursing selected, with the moral and material advantages of the life. 4. Intending competitors must send their names and addresses to the Editor of THE HOSPITAL, 28 Southampton Street, Strand, W.C. 2, by Sept. 1; the leaflets must reach him at the same address not later than Saturday, September 28, 1918. 5. Provided that thirty competitors enter and thirty leaflets, in sets of three, at least are received, three prizes will be given of £3 3s., £2 2s., and £1 1s.

---

**☞ IT is our invariable practice to pay for all items of news which we may publish. The minimum payment is 5s. Paragraphs of about twenty lines in length—that is to say, of 150 words or less—are preferred. Contributions should be addressed to The Editor, THE HOSPITAL, 28 and 29 Southampton Street, Strand, London, W.C. 2, and, to be in time for the current week's issue, should reach him by the first post on Mondays.**

